# Point prevalence survey of antibiotic use in hospitals in Latin American countries

**DOI:** 10.1093/jac/dkab459

**Published:** 2021-12-27

**Authors:** Gabriel Levy Hara, Robin Rojas-Cortés, Helvert Felipe Molina León, Anahí Dreser Mansilla, Ismary Alfonso Orta, José Noe Rizo-Amezquita, René Guillermo Santos Herrera, Silvia Mendoza de Ayala, Marlen Arce Villalobos, Hilda Mantilla Ponte, Ever Davila, Gloria Aguilar, Analía Porrás, Pilar Ramón-Pardo, José Luis Castro, on behalf of the Latin American Point Prevalent Survey Study Group, Daniela Guzmán, María Luisa Rioseco, Jaime Labarca, José Pablo Díaz Madriz, Josué Murillo Cubero, Allan Robles Calderón, Mónica Alfaro, Luisa Arias Soto, Alejandro Aayon, Tania Jiménez Oreamuno, Zulema Jiménez, Adriana Sequeira, Jorge Mederos Hernández, Jorge Luis Campistrous Lavaut, Damarys Castillo Meriño, Elsa Fleitas Ruisanchez, Damaris Portuondo Sánchez, Humberto Guanche Garcell, Juan José Pisonero Socias, Evelyn Perera Díaz, Norma America Cardoso Lunar, Irene Fiterre Lancis, José Antonio Álvarez Ramírez, Midsay López Leyte, Ariadna Méndez Rosabal, Ebel Aldana Estrada, Mariela Cano, Leonel Méndez, Anay Cordero Eiriz, Liana Padrón Menéndez, Gladys Fuentes Fernández, Raunel Reyes Ayala, Salomón Monroy, Ramón Menjívar, Carmen Elena Albanez Martínez, Diana Cabrera, Sofía Mercedes Menjivar Delgado, Gustavo Antonio Molina Guzmán, Rafael Mejía, Carolina Rodríguez, Sara Alvarenga, Mirian Alvarado, Ruth del Carmen Alvarado de Zelaya, Germán Arévalo, Guillermo Parada, Luis Cuellar, Alexis Holguín Ruiz, Yuan Almora Pinedo, Eduardo Sánchez Vergaray, Luis Enrique Vasquez Gil, Bertha Gizel Injante Ibazeta, Esther Dina Guadalupe Ricra, Rony Estrada Segura, Coralith García Apac, Jennifer Cuadros Inga, Roger Hernández Díaz, Marie Vallejo Vigo, Cesar Mujica Cuba, Pierina Vilcapoma, Eddie Angles-Yanqui, Débora Rocío Mananita Terrones, Rosa Terán Robles, Cristhian Resurrección, Alfredo Chiappe Gonzalez, Martha Antezana, Socorro Torres Zegarra, Miguel Villegas-Chiroque, Roberto Díaz-Sipión, Jorge Benítez-Peche, Jorge Luis Alave Rosas, Jhoselyn Laura Goytizolo Ruiz, Angélica María Hernández Fernández, José Antonio Flores Vargas, Javier Araujo, Francisco Javier Arriaga García, Zoila Cruz Rivera, Víctor Monroy Colín, Katia Bustamante Ríos, Jorge Israel Hernández Blanquel, Eduardo Arias de la Garza, Héctor Hernández Gutiérrez, Karla León, Hortencia Esther Peralta Lara, Carlos Baltodano Arias, Eduardo Alemán Garay, Gladys Estigarribia Sanabria, Livio Mereles Menchaca, Natalia Luraschi Viré, Dasy Acuña, Edgar Giménez Caballero

**Affiliations:** Hospital Durand, Buenos Aires, Argentina; Pan American Health Organization/World Health Organization, Washington, DC, USA; Pan American Health Organization/World Health Organization, Washington, DC, USA; Instituto de Salud Pública, Cuernavaca, México; Centro para el Control Estatal de Medicamentos, Equipos y Dispositivos Médicos, Ciudad Habana, Cuba; Comisión Nacional de Arbitraje Médico, Ciudad de México, México; Dirección Nacional de Enfermedades Infecciosas, Ministerio de Salud, San Salvador, El Salvador; Instituto Salvadoreño del Seguro Social, San Salvador, El Salvador; Dirección Vigilancia de la Salud, Ministerio de Salud, San José, Costa Rica; Dirección General de Medicamentos, Insumos y Drogas, Ministerio de Salud, Lima, Perú; División General de Insumos Médicos, Ministerio de Salud, Managua, Nicaragua; Instituto Regional de Investigación en Salud, Universidad Nacional de Caaguazú, Coronel Oviedo, Paraguay; Pan American Health Organization/World Health Organization, Washington, DC, USA; Pan American Health Organization/World Health Organization, Washington, DC, USA; Pan American Health Organization/World Health Organization, Washington, DC, USA

## Abstract

**Background:**

Point prevalence surveys (PPSs) on antibiotic use are useful for understanding different aspects related to prescription patterns in hospitals.

**Methods:**

An adaptation of the WHO methodology for a PPS on antibiotic use was applied. Hospital wards were divided into medical (MED), surgical (SUR), ICUs, gynaecology and obstetrics (GO), high-risk (HR) and mixed wards (MIX). A web application (RedCap^©^) through a mobile device was used for data collection.

**Results:**

Between December 2018 and August 2019, 5444 patients in 33 hospitals in five countries were included (10 hospitals in Cuba, 7 in Paraguay, 6 in El Salvador, 5 in Mexico and 5 in Peru). Of these patients, 54.6% received at least one antibiotic, with variations between and within hospitals and countries. Antibiotics were more frequently used in ICUs (67.2%), SUR (64.5%) and MED wards (54.2%), with 51.2% of antibiotics prescribed for community-acquired infections (CAIs), 22.9% for healthcare-associated infections (HAIs), 11.1% for surgical prophylaxis and 6.1% for unknown reasons. Adherence to guidelines was observed in 68.6% of cases (72.8% for CAIs, 72.4% for HAIs and 44.3% for prophylaxis). Third-generation cephalosporins were the class of antibiotics most frequently used (26.8%), followed by carbapenems (10.3%) and fluoroquinolones (8%). Targeted treatments were achieved in 17.3% of cases.

**Conclusions:**

Antibiotic use was generally higher than that published in other studies. There is an urgent need to promote and strengthen the antimicrobial stewardship programmes in Latin America.

## Introduction

Antimicrobial resistance (AMR) is a worldwide phenomenon that has worsened in recent decades, linked to the increased use and abuse of antimicrobials, which have spread not only in human and veterinary medicine, but also to other fields such as agriculture and the environment.^1^

Countries in the Americas began the implementation of National Action Plans in line with the WHO Global Action Plan on Antimicrobial Resistance launched in 2015.^2^ A key action is to optimize the use of antimicrobials in human and animal health, addressing the need to implement antimicrobial stewardship programmes (ASPs) both in hospitals and primary care settings.

Surveillance systems of antimicrobial consumption and AMR provide essential data for implementing ASPs. Continuous data collection on antibiotic prescribing is not easy due to the high workload and level of resources needed.^3^ A viable alternative is to collect data at a specific point in time, which can be done by using the point prevalence survey (PPS) methodology. This type of survey permits (i) the measurement of antimicrobial use along time, assessing changes in prescribing trends; (ii) the identification of targets for quality improvement in different hospital wards; and (iii) the evaluation of the effectiveness of interventions implemented in response to indicators identified during previous surveys.^4^ PPSs have been used in global^5^ and regional studies in the Caribbean^6^ and Europe,^7^ as well as in country-level studies in China,^8^ Saudi Arabia,^9^ the USA^10^ and Viet Nam,^11^ among others.^12–14^

Antimicrobial consumption studies in hospitals from countries in Latin America are scarce. A recently published scoping review on ASPs in Latin America showed that although utilization of antimicrobials was the most frequently reported outcome, most studies had been done by measuring DDDs and only a few through a PPS.^15^ During recent years, multicountry studies, such as the Global-PPS of antimicrobial consumption,^5^ which included 4122 patients from 21 hospitals in Argentina, Chile, Costa Rica and Brazil, and a PPS on healthcare-associated infections (HAIs) and antimicrobial consumption including 2740 patients from 11 hospitals from four Latin American countries—Brazil, Colombia, Mexico and Venezuela—were conducted.^16^

The present Latin American PPS (Latin-PPS) began with an initial pilot phase conducted between November 2017 and February 2018, including 12 hospitals from four countries: Costa Rica (4), Peru (3), Chile (2) and Nicaragua (2) (unpublished data). After this pilot phase, some improvements, mostly related to data collection and analysis, were introduced.

This article presents results of the Latin-PPS carried out after the pilot phase in 33 Latin American hospitals from five countries (Cuba, El Salvador, Mexico, Paraguay and Peru) as a baseline survey to implement or strengthen existing ASPs.

## Methods

The Latin-PPS included minor adaptations of the WHO methodology for a PPS on antibiotic use in hospitals. As in the WHO protocol, only antibiotics were considered (see below). Main differences were the exclusion of the McCabe score, and differences in the criteria to assess compliance with clinical practice guidelines (CPGs). All variables collected are shown in Appendix 1, available as Supplementary data at *JAC* Online.^3^ The following is a summary of the main methodological aspects.

### Hospital selection

A sample of hospitals was selected by the Ministries of Health (MoHs) in agreement with the Latin-PPS coordination team and designated partners (such as universities) according to some predefined criteria (e.g. hospital size, regional distribution, feasibility, human resources potentially involved and needs and interest in implementing or strengthening an ASP in the near future).

### Patient selection

All patients hospitalized according to the daily census in the ward at 8:00 a.m. on the day of the study were included in the survey, regardless of whether they were receiving antibiotics or not. Day-case patients (e.g. those undergoing same-day treatment or surgery and discharge, outpatient departments, emergency room or outpatient dialysis) were excluded.

### Survey procedures

All beds in each ward (e.g. general surgery) were surveyed in a single day, and each ward was studied only once during the period. Prior to starting the study, hospital coordinators were asked to submit the schedule listing which wards would be surveyed each day and the number of researchers to be deployed. This aimed to assess the feasibility of conducting the study in an organized way. Data collection for the entire hospital was completed within a maximum of three consecutive weeks from the first day of data collection.

Hospital wards were divided into medical (MED), surgical (SUR), ICUs (including medical, surgical, paediatric and neonatal units), gynaecology and obstetrics (GO), high-risk (HR; haematology, oncology, burns, transplantation and infectious diseases) and mixed (MIX) wards. The latter consisted of units where patients were admitted without differentiation between medical and surgical diseases.

### Contents of the survey

The survey was divided into two sections. The first one (patient information) needed to be completed for all admitted patients, and included the type of ward, demographics, date of admission, catheterizations, intubation and surgery during the current admission. The second part (indication and antibiotics data) needed to be completed only for patients receiving oral or parenteral antibiotics on the day of the survey. Antibiotics previously prescribed during admission were excluded. All systemic antibiotics listed in the original WHO protocol (ATC codes J01) plus oral presentation of vancomycin (ATC AO7A) and metronidazole (P01AB01) were available to be ticked in a dropdown list. Topical antibiotics and those used for the treatment of tuberculosis were excluded. The information requested in this section included the type of indication (treatment or prophylaxis), guidance for treatment (empiric or tailored to microbiological findings), diagnosis, microbiological results, antibiotics prescribed (drug, dose, interval, route of administration) and compliance with CPGs. A prescription was considered compliant if it was in line with local, national or international CPGs in use at the institution, as defined by the research team. When assessment of compliance was not possible (e.g. type of indication unknown or other than prophylaxis or treatment; diagnosis unknown or undefined), it was classified as not assessable.

### Training

Virtual sessions were held for coordinators and investigators from each hospital. These included a practical revision of study variables and information technology aspects, followed by simulation exercises based on current real cases to adjust all proceedings.

### Data collection and review

Data were directly uploaded to RedCap^©^, a tool that includes a mobile app functionality that allows offline data collection on tablets and smartphones. Electronic forms were formatted to include multiple quality control checks to avoid wrong data entry.

Patient identities were known only by local researchers, and patient information was uploaded anonymously, through a previously assigned code for each unit and hospital. Throughout the study period, study coordinators reviewed all files between 24 and 72 h after being uploaded, allowing prompt detection of missing data (e.g. age, gender, date of admission, type of indication, diagnosis) and inconsistencies. This review made it possible to quickly hold discussions with the local coordinators, verify quality of data and use standardized and homogenized criteria, especially to determine adherence to CPGs. Finally, in less than 5% of the patients, some type of data (mainly type of indication or diagnosis) was not available for analysis. Data were safely stored in a server hosted by the Pan American Health Organization (PAHO).

### Data analysis

The analysis was performed using the R software environment. Absolute frequencies and proportions are reported for qualitative variables. Means and ranges are presented for continuous variables. Data from individual hospitals were aggregated to calculate all indicators.

As the present study was not conducted on a random sample of hospitals, no countrywide inference measures were calculated. Therefore, statistical tests were deemed not to be required, as the analysis was limited to the sample of hospitals included. At the hospital level, no inference was necessary as a daily inpatient census had been included during the study period.^17^^,^^18^

### Ethics

The study was approved by the Ethics Committee for each hospital in Cuba, Mexico, Paraguay and Peru, as well as by the PAHO Ethics Review Committee. In the case of El Salvador, approval was provided by the National Committee for Ethics of Research in Health.

## Results

The Latin-PPS was conducted between December 2018 and August 2019 and included 33 hospitals from five countries: Cuba (10 hospitals), Paraguay (7), El Salvador (6), Mexico (5) and Peru (5). A total of 5444 patients were surveyed, with a mean of 165 patients (range 22–469) per hospital, higher for El Salvador’s hospitals (mean 326.2 participants per hospital) and lower for Paraguay (mean 63.8). Eighteen (55%) hospitals had more than 200 beds, 10 (30%) between 100 and 200, and 5 (15.1%) had fewer than 100 beds. Thirty facilities belonged to the public sector (90.9%) and 24 were located in the capital city (72.7%). The average occupancy rate during the study period was 60%, except for Paraguay (48.7%). The main reason for this relatively low bed occupancy was that the study covered two vacation periods (December–February and July–August) with the consequent reduction in hospitalization. Regarding their complexity, 16 were tertiary hospitals (48.5%), 10 were secondary (30.3%), 6 were specialized (18.2%) and 1 was for primary care (3%). Mean age of participants was similar for all countries (42.7 years; range 0–102); 4376 (80.4%) of patients were ≥18 years old. Table 1 shows characteristics of hospitals and Table 2 the demographic information of patients included.

**Table 1.** Characteristics of hospitals included in the Latin-PPS, 2018–19

Characteristic	Cuba	Mexico	El Salvador	Peru	Paraguay	Total
Number of hospitals included	10	5	6	5	7	33
Number of participants	1197	585	1957	1258	447	5444
Average number of participants by hospital (range)	119.7 (22–306)	117 (31–213)	326.2 (181–469)	251.6 (97–391)	63.8 (37–98)	165.0 (22–469)

**Table 2.** Demographics of patients included in the Latin-PPS, 2018–19

Demographic	Cuba	Mexico	El Salvador	Peru	Paraguay	Total
Mean age (years)	46.7	36.7	44.4	41.9	34.4	42.7
Age categories, years, *n* (%)						
<1	62 (5.2)	72 (12.3)	148 (7.6)	162 (12.9)	62 (13.9)	506 (9.3)
1–4	60 (5.0)	25 (4.3)	60 (3.1)	36 (2.9)	31 (6.9)	212 (3.9)
5–17	73 (6.1)	67 (11.5)	111 (5.7)	54 (4.3)	45 (10.1)	350 (6.4)
18–65	634 (52.9)	317 (54.2)	1126 (57.5)	695 (55.2)	230 (51.5)	3002 (55.2)
>65	368 (30.7)	104 (17.8)	512 (26.2)	311 (24.7)	79 (17.7)	1374 (25.2)
Gender						
Male	576 (48.1)	321 (54.9)	953 (48.7)	570 (45.3)	238 (53.2)	2658 (48.8)
Female	621 (51.9)	264 (45.1)	998 (51.0)	686 (54.5)	207 (46.3)	2776 (51.0)
Transgender	0 (0)	0 (0)	2 (0.10)	1 (0.08)	1 (0.2)	4 (0.07)
Unknown	0 (0)	0 (0)	4 (0.20)	1 (0.08)	1 (0.2)	6 (0.11)

Fifty-four percent of patients received at least one antibiotic, with considerable variations between and within hospitals and countries (Table 3). Ten percent of treatments were administered orally (varying from 5.7% in Mexico to 12.9% in Cuba). The lowest antibiotic use was found in Cuban hospitals (47.6%) and the highest in the Paraguayan sample (81.1%). In general, ICUs had the highest prevalence of antibiotic use (67.2%), ranging from 44.5% in Peru to 83.9% in El Salvador. SUR wards (64.5%) had the second highest prevalence (ranging from 56.8% in Peru to 84.4% in Paraguay), followed by MED wards (54.2%) (ranging from 48.2% in Cuba to 79.3% in Paraguay). Antibiotic use in adult units was 52.1% and in paediatric units was 58.8%.

**Table 3.** Prevalence of antibiotic use by country and type of ward in the Latin-PPS, 2018–19

	Cuba		Mexico		El Salvador		Peru		Paraguay		Total	
	Admitted, *n*	Antibiotic use, *n* (%)	Admitted, *n*	Antibiotic use, *n* (%)	Admitted, *n*	Antibiotic use, *n* (%)	Admitted, *n*	Antibiotic use, *n* (%)	Admitted, *n*	Antibiotic use, *n* (%)	Admitted, *n*	Antibiotic use, *n* (%)
Prevalence of antibiotic use^a^	1197	570 (47.6)	584^b^	359 (61.5)	1957	1076 (55)	1258	604 (48.0)	446^b^	362 (81.1)	5442^b^	2971 (54.6)
Prevalence of antibiotic use by ward type												
MED	556	268 (48.2)	250	145 (58.0)	1182	626 (53.0)	767	392 (51.1)	242	192 (79.3)	2997	1623 (54.2)
SUR	193	123 (63.7)	214	146 (68.2)	330	211 (63.9)	220	125 (56.8)	64	54 (84.4)	1021	659 (64.5)
ICUs**c**	96	68 (70.8)	64	42 (65.6)	93	78 (83.9)	92	41 (44.5)	33	25 (75.7)	378	254 (67.2)
GO	148	46 (31.1)	31	11 (35.5)	166	82 (49.4)	109	29 (26.6)	107	91 (85.0)	561	259 (46.2)
HR	152	52 (34.2)	25	15 (60.0)	158	61 (38.6)	17	1 (5.8)	0	0 (0)	352	129 (36.6)
MIX	52	13 (25)	0	0 (0)	28	18 (64.3)	53	16 (5.8)	0	0 (0)	133	47 (35.3)

a Number of patients who received at least one antibiotic out of the total number of hospitalized patients.

b One registry in Mexico and one in Paraguay were missing one type of ward variable.

c Adult, paediatric and neonatal ICUs.

Overall, community-acquired infections (CAIs) were the most frequent reason for prescribing antibiotics (51.2%), followed by HAIs (22.9%), surgical prophylaxis (11.1%) and medical prophylaxis (4.0%). In 6.1% of the cases, the reason (e.g. CAI, HAI, prophylaxis) was not stated in the medical record; in 4.7% of cases, antibiotics were prescribed for other situations not related to treatment or prophylaxis, where antibiotics are typically not indicated (e.g. tumours, trauma, closed fractures, stroke, gastrointestinal bleeding, cholelithiasis) (Table 4).

**Table 4.** Distribution of antibiotic use by type of indication in the Latin-PPS, 2018–19

Distribution by antibiotic indication type^a^	Cuba	Mexico	El Salvador	Peru	Paraguay	Total
	*n* (%)	*n* (%)	*n* (%)	*n* (%)	*n* (%)	*n* (%)
HAI	119 (20.3)	98 (26.5)	283 (25.3)	165 (26.5)	35 (9.5)	700 (22.9)
CAI	334 (56.9)	152 (41.1)	527 (47.3)	356 (57.2)	197 (53.7)	1566 (51.2)
Medical prophylaxis	17 (2.9)	9 (2.4)	41 (3.8)	15 (2.4)	41 (11.2)	123 (4.0)
Surgical prophylaxis	77 (13.0)	43 (11.6)	110 (10.0)	45 (7.2)	64 (17.4)	339 (11.1)
Other^b^	5 (0.9)	38 (10.3)	67 (6.1)	15 (2.4)	18 (4.9)	143 (4.7)
Unknown^c^	35 (6.0)	30 (8.1)	84 (7.5)	27 (4.3)	12 (3.3)	188 (6.1)
Total number of indications	587	370	1112	623	367	3059

a Number of this specific type of indication (e.g. HAI, CAI, surgical prophylaxis) out of the total number of indications. Each patient could have up to three indications, so that the total number of indications exceeds the number of patients surveyed in each country indicated in Table 3.

b Other includes situations not related to treatment or prophylaxis, where antibiotics are typically not indicated (e.g. tumours or cancer, trauma, closed fractures, stroke or vascular neurological sequelae, cirrhosis, gastrointestinal bleeding, cholelithiasis, nephrolithiasis, lung bullae, dialysis, uncomplicated pancreatitis, pancytopenia, uninfected diabetic foot, deep vein thrombosis, morbid obesity, pneumothorax, non-specific pleural effusion, uncomplicated postpartum period).

c Type of indication (e.g. HAI, CAI or prophylaxis) unknown.

Main diagnoses were similar among countries, pneumonia being the most frequent (26.4%), followed by urinary tract infections (15.3%), non-surgical infections involving skin or soft tissue (12.7%), intra-abdominal, excluding gastrointestinal infections (11.7%) and clinical sepsis (7%) (Table 5).

**Table 5.** Diagnoses for which antibiotics for treatment were prescribed in the Latin-PPS, 2018–19

Diagnosis^a^	Cuba	Mexico	El Salvador	Peru	Paraguay	Total
	*n* ^b^ (%)	*n* ^b^ (%)	*n* ^b^ (%)	*n* ^b^ (%)	*n* ^b^ (%)	*n* ^b^ (%)
PNEU	158 (35.0)	57 (23.2)	214 (25.8)	105 (20.4)	64 (28.4)	598 (26.4)
SST-O	69 (15.3)	19 (7.7)	96 (11.6)	76 (14.8)	28 (12.4)	288 (12.7)
IA	30 (6.7)	41 (16.7)	88 (10.6)	72 (14.0)	33 (14.7)	264 (11.7)
CYS	13 (2.9)	18 (7.3)	91 (11.0)	35 (6.8)	18 (8.0)	175 (7.7)
PYE	54 (12.0)	22 (8.9)	49 (5.9)	44 (8.6)	3 (1.3)	172 (7.6)
CSEP	28 (6.2)	29 (11.8)	36 (4.3)	49 (9.5)	18 (8.0)	160 (7.1)
SST-SSI	25 (5.5)	13 (5.3)	71 (8.6)	22 (4.3)	9 (4.0)	140 (6.2)
BAC	10 (2.2)	12 (4.9)	26 (3.1)	14 (2.7)	4 (1.8)	66 (2.9)
GI	10 (2.2)	5 (2.0)	29 (3.5)	17 (3.3)	5 (2.2)	66 (2.9)
BRON	11 (2.4)	3 (1.2)	20 (2.4)	10 (2.0)	7 (3.1)	51 (2.3)
OBGY	6 (1.3)	2 (0.8)	14 (1.7)	16 (3.1)	13 (5.8)	51 (2.3)
ENT	21 (4.7)	4 (1.6)	9 (1.1)	7 (1.4)	4 (1.8)	45 (2.0)
FN	5 (1.1)	11 (4.5)	19 (2.3)	8 (1.6)	0 (0)	43 (1.9)
BJ-O	2 (0.4)	1 (0.4)	14 (1.7)	15 (2.9)	8 (3.6)	40 (1.8)
CNS	6 (1.3)	2 (0.8)	9 (1.1)	10 (2.0)	6 (2.7)	33 (1.5)
BJ-SSI	1 (0.2)	6 (2.4)	14 (1.7)	4 (0.8)	1 (0.4)	26 (1.2)
ASB	0 (0)	0 (0)	13 (1.6)	9 (1.8)	2 (0.9)	24 (1.1)
CVS	2 (0.4)	1 (0.4)	4 (0.5)	1 (0.2)	1 (0.4)	9 (0.4)
GUM	0 (0)	0 (0)	7 (0.8)	0 (0)	1 (0.4)	8 (0.4)
EYE	0 (0)	0 (0)	7 (0.8)	0 (0)	0 (0)	7 (0.3)
TOTAL	451	246	830	514	225	2266

a PNEU, pneumonia; SST-O, cellulitis, wound, deep soft tissue not involving bone, not related to surgery; IA, intra-abdominal sepsis, including hepatobiliary; CYS, symptomatic lower urinary tract infection; PYE, symptomatic upper urinary tract infection; CSEP, clinical sepsis, suspected bloodstream infection without lab confirmation/results not available, no blood cultures collected or negative blood culture, excluding febrile neutropenia; SST-SSI, surgical site infection involving skin or soft tissue but not bone; UND, completely undefined; site with no systemic inflammation; BAC, laboratory-confirmed bacteraemia; GI, gastrointestinal infections; OBGY, obstetric or gynaecological infections, sexually transmitted infections in women; BRON, acute bronchitis or exacerbations of chronic bronchitis; ENT, infections of ear, nose, throat, larynx and mouth; FN, febrile neutropenia or other form of manifestation of infection in immunocompromised host (e.g. HIV, chemotherapy) with no clear anatomical site; BJ-O, septic arthritis, osteomyelitis, not related to surgery; CNS, infections of the CNS; BJ-SSI, septic arthritis, osteomyelitis of surgical site; ASB, asymptomatic bacteriuria; CVS, cardiovascular infections (endocarditis, vascular graft); GUM, prostatitis, epididymo-orchitis, sexually transmitted infections in men; EYE, endophthalmitis.

b Number of patients who received antibiotics to treat each specific infection (e.g. pneumonia, cellulitis) out of the total number of patients treated for any infection. Patients with unknown diagnosis or who received antibiotics for prophylaxis are excluded.

Overall, 68.6% of assessable prescriptions were considered compliant with CPGs, ranging from 61% in Paraguay to 72.6% in Peru (Table 6). Adherence to CPGs was higher in HR, MIX and MED wards than in ICUs, GO and SUR wards, with some differences between countries. Compliance was higher for treatment (around 72%) than for prophylaxis (44.3%). The main reason for non-compliance in surgical prophylaxis was its duration for more than 24 h in 58% of cases, ranging from 46% in Cuba to 60% in Peru.

**Table 6.** Compliance with guidelines for treatment and prophylaxis in the Latin-PPS, 2018–19^a^

	Cuba	Mexico	El Salvador	Peru	Paraguay	Total
	*n*/*N* (%)	*n*/*N* (%)	*n*/*N* (%)	*n*/*N* (%)	*n*/*N* (%)	*n*/*N* (%)
Guideline compliance^b^	457/695 (65.7)	321/442 (72.6)	927/1332 (69.6)	622/873 (71.2)	291/474 (61.0)	2618/3816 (68.6)
Guideline compliance by indication type						
HAI	108/161 (67.1)	126/145 (86.9)	295/420 (70.2)	188/267 (70.4)	42/56 (75)	760/1049 (72.4)
CAI	297/419 (70.9)	175/227 (77.1)	544/749 (72.6)	411/531 (77.4)	191/296 (64.5)	1618/2222 (72.8)
Antibiotic prophylaxis	52/115 (45.2)	20/70 (28.5)	88/163 (54.0)	22/75 (29.3)	58/126 (46.0)	240/545 (44.3)
Guideline compliance by ward type						
MED	245/343 (71.4)	140/170 (82.3)	583/791 (73.7)	410/557 (73.6)	169/271 (47.6)	1547/2102 (73.6)
SUR	70/145 (48.3)	128/196 (65.3)	141/241 (58.5)	115/176 (65.3)	43/65 (66.1)	497/823 (60.4)
ICUs	53/94 (56.4)	29/45 (64.4)	56/102 (54.9)	52/79 (65.8)	25/40 (62.5)	215/360 (59.7)
GO	38/69 (55.1)	9/12 (75.0)	72/98 (73.5)	25/37 (67.5)	54/98 (55.1)	198/314 (63.0)
HR	41/59 (69.5)	15/19 (63.1)	61/78 (78.2)	1/1 (100)	0 (0)	118/157 (75.1)
MIX	10/15 (66.7)	0 (0)	14/22 (63.6)	19/23 (82.6)	0 (0)	43/60 (71.6)

a Other and unknown indications (as described in Table 4) are excluded, due to the lack of reliability in establishing compliance with guidelines.

b Number of antibiotics prescribed according to guidelines out of the total number of antibiotics prescribed for this specific type of indication or ward (as applicable).

Third-generation cephalosporins (3GCs) was the class most frequently used (26.8%), followed by carbapenems (10.3%), fluoroquinolones (8%), metronidazole (7.6%) and vancomycin (6.7%) (Table 7). Carbapenems were most frequently used in Mexico and Peru, and less frequently prescribed in Cuba and Paraguay. Cuban hospitals also had a lower use of glycopeptides, and Paraguayan hospitals a lower use of 3GCs. Globally, penicillins plus a β-lactam inhibitor represented 5.6% of total antibiotics prescribed.

**Table 7.** Antibiotics prescribed in the Latin-PPS, 2018–19

Antibiotic group	Cuba	Mexico	El Salvador	Peru	Paraguay	Total
	*n* ^a^ (%)	*n* (%)	*n* (%)	*n* (%)	*n* (%)	*n* (%)
J01DD 3GCs (ceftriaxone, cefotaxime, ceftazidime)	223 (29.8)	132 (24.9)	468 (29.9)	233 (24.8)	95 (18.3)	1151 (26.8)
J01DH Carbapenems (meropenem, imipenem, ertapenem)	20 (2.7)	80 (15.1)	164 (10.5)	159 (17.0)	21 (4.1)	444 (10.3)
J01MA Fluoroquinolones (ciprofloxacin, levofloxacin)	52 (6.9)	44 (8.3)	147 (9.4)	58 (6.2)	44 (8.5)	345 (8.0)
J01XD Imidazole derivatives (metronidazole)	77 (10.3)	44 (8.3)	122 (7.8)	56 (6.0)	30 (5.8)	329 (7.6)
J01XA Glycopeptide antibacterials (vancomycin)	25 (3.3)	50 (9.4)	96 (6.1)	94 (10.0)	25 (4.8)	290 (6.7)
J01GB Other aminoglycosides (amikacin, gentamicin)	42 (5.6)	37 (7.0)	106 (6.8)	59 (6.3)	27 (5.2)	271 (6.3)
J01FF Lincosamides (clindamycin)	7 (0.9)	27 (5.1)	92 (5.9)	87 (9.3)	35 (6.8)	248 (5.8)
J01CR Combinations of penicillins, including β-lactamase inhibitors (amoxicillin/sulbactam, piperacillin/ tazobactam, ampicillin/sulbactam, amoxicillin/clavulanic acid)	24 (3.2)	19 (3.6)	93 (5.9)	21 (2.2)	86 (16.6)	243 (5.6)
J01CA Penicillins with extended spectrum (ampicillin, amoxicillin)	5 (0.7)	26 (4.9)	96 (6.1)	23 (2.5)	62 (12.0)	212 (4.9)
J01DB First-generation cephalosporins (cefazolin, cefalotin)	41 (5.5)	21 (4.0)	47 (3.0)	39 (4.2)	39 (7.5)	187 (4.3)
J01DC Second-generation cephalosporins (cefuroxime)	106 (14.2)	1 (0.2)	0 (0)	6 (0.6)	0 (0)	113 (2.6)
J01EE Combinations of sulphonamides and trimethoprim, including derivatives (trimethoprim/sulfamethoxazole)	34 (4.5)	9 (1.7)	18 (1.1)	25 (2.7)	1 (0.2)	87 (2.0)
J01FA Macrolides (azithromycin, clarithromycin)	22 (2.9)	3 (0.6)	16 (1.0)	19 (2.0)	12 (2.3)	72 (1.7)
J01DE Fourth-generation cephalosporins (cefepime)	37 (4.9)	13 (2.5)	15 (1.0)	3 (0.3)	0 (0)	68 (1.6)
J01DB First-generation cephalosporins (cefalexin)	11 (1.5)	3 (0.6)	0 (0)	4 (0.4)	30 (5.8)	48 (1.1)
J01AA Tetracyclines (doxycycline)	0 (0)	7 (1.3)	17 (1.1)	4 (0.4)	1 (0.2)	29 (0.7)
J01CF β-Lactamase-resistant penicillins (oxacillin)	0 (0)	0 (0)	12 (0.8)	15 (1.6)	2 (0.4)	29 (0.7)
J01CE β-Lactamase-sensitive penicillins (penamecillin)	0 (0)	0 (0)	24 (1.5)	0 (0)	0 (0)	24 (0.6)
J01XB Polymyxins (colistin)	5 (0.7)	0 (0)	0 (0)	13 (1.4)	0 (0)	18 (0.4)
J01XE Nitrofuran derivatives (nitrofurantoin)	1 (0.1)	0 (0)	14 (0.9)	0 (0)	0 (0)	15 (0.3)
Other antibiotics	17 (2.3)	14 (2.6)	20 (1.3)	20 (2.1)	8 (1.5)	79 (1.8)
Total	749	530	1567	938	518	4302

a Total number of antibiotics included in the study prescribed; some patients received more than one antibiotic for treatment of surgical prophylaxis.

**Figure 1.**
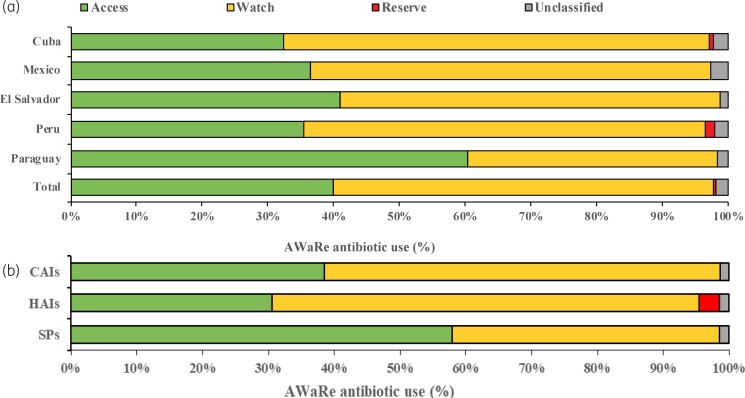
Percentage of antibiotic use according to the WHO AWaRe classification. (a) Percentage of overall use by country. (b) Percentage of antibiotic use according to the type of indication. SPs, surgical prophylaxis. This figure appears in colour in the online version of *JAC* and in black and white in the print version of *JAC*.

Figure 1 shows patterns of antibiotic use according to the WHO Access, Watch and Reserve (AWaRe) classification. The highest proportion corresponded to the Watch group (57.7%), followed by the Access group (40%) and then the Reserve group (0.4%). Access was the most frequently used group for surgical prophylaxis (57.9%), while antibiotics belonging to the Watch group prevailed for the treatment of CAIs (60.1%) and HAIs (64.8%).

Regarding the type of indication, 3GCs were the class mainly prescribed for CAIs (30.6%) followed by fluoroquinolones (9.7%), carbapenems (8.4%) and metronidazole (8.2%) (Table S1); carbapenems (21.4%), glycopeptides (16.3%) and 3GCs (16%) were the antibiotics most frequently prescribed for treating HAIs (Table S2). For surgical prophylaxis, first-generation cephalosporins accounted for 35.7% of all prescriptions, and 3GCs represented 29.1% of all antibiotics prescribed, reaching 52.9% in the sample from El Salvador (Table S3).

Overall, microbiological studies were requested in 44.3% prior to starting antibiotic treatment, with Cuban (19.6%) and Paraguayan (27.6%) hospitals showing the lowest figures. Targeted treatments were achieved in 17.3% of cases, being higher for Mexican hospitals (27.4%) (Table S4).

## Discussion

The present Latin-PPS showed that more than half of hospitalized patients received an antibiotic on the day of the survey. Considering exclusively the use of antibiotics (excluding all other antimicrobials), the prevalence for the Latin American countries studied was higher than that reported in the Global-PPS in 2015, conducted only in adults (around 31%),^5^ the European PPS in 2016–17 (28%),^7^ the USA PPS in 2015 (46%)^10^ and the Saudi Arabia PPS (44%),^9^ but similar to that from China (56%)^8^ and lower than that from Viet Nam (67.4%).^11^ Even those European countries with higher use (e.g. Cyprus, Bulgaria, Italy, Malta and Spain)^8^ exhibited a prevalence of 40%–45%, lower than the figures found in the present study.

The prevalence of antibiotic use found in this research was similar to that reported in the previous Latin American study (50%),^16^ but higher than that found in Latin America at the Global-PPS (33.3%).^5^ Although the Latin-PPS included 5444 patients from 33 hospitals in five countries, compared with 4122 patients from 19 hospitals in four countries in the Global-PPS, sample size alone wouldn’t justify these differences. Instead, they might be due to the fact that other countries were included in the Global-PPS (Argentina, Brazil, Chile and Costa Rica), the characteristics of hospitals involved, and other factors such as the temporality of data collection. Therefore, comparisons among different PPS studies should be made with caution and considering contextual information.

The higher prevalence of use of antibiotics in ICUs is consistent with previous publications.^5^^,^^8^^,^^10^^,^^11^

In general, the prescription of 3GCs and carbapenems in this sample of hospitals from Latin America was higher than in most studies.^5^^,^^7^^,^^10^ Compared with the earlier study in Latin America,^16^ the use of 3GCs was also higher, but that of carbapenems was similar. The frequent use of 3GCs and carbapenems has also been reported from single-hospital studies in Peru^19^ and Mexico.^20^ Regarding the treatment of HAIs, 3GCs represented 16% of antibiotics used, higher than in Central Europe (2%–10%),^5^^,^^7^ the USA (7%)^5^ and Asia (10%–13%),^5^ but lower than in Eastern Europe (20%).^5^ Similarly, carbapenems were prescribed in 21% of cases, comparable with the 18%–20% in Asia, but higher than in Europe (8%–16%, depending on the region)^5^^,^^7^ and the USA (7%).^5^

Potential differences of resistance in Gram-negative bacilli could partly contribute to these prescription patterns. According to the SENTRY antimicrobial surveillance programme, the detection of an ESBL gene among non-carbapenem-resistant *Escherichia coli* and *Klebsiella pneumoniae* was 8.2% in Europe, 15.4% in Asia-Pacific and 30.3% in Latin America.^21^ In turn, limited available data suggest that the prevalence of carbapenem-resistant *E. coli, K. pneumoniae, Acinetobacter baumannii* and *Pseudomonas aeruginosa* might be similar across regions.^22^ Hence, the apparently higher prevalence of ESBLs may in part justify the increased use of some broader-spectrum antibiotics, such as carbapenems, and lower use of piperacillin/tazobactam in Latin America. Additionally, the low prescription of ampicillin/sulbactam, amoxicillin/clavulanate, amoxicillin/sulbactam, doxycycline and macrolides was probably also related to resistance patterns. However, other key drivers such as cultural habits and poor awareness, understanding and training on the AMR problem^23^ are likely to have a much greater influence on prescribing patterns.

Overall adherence to CPGs was found in about two-thirds of the cases, considerably better when antibiotics were prescribed for therapy than for prophylaxis. Specifically, regarding surgical prophylaxis, both the excessive use of ceftriaxone and the prolongation of surgery beyond 24 h have also been observed in several regions of the world.^5^ As described above, this assessment was initially done by the local team, and discussed with the study coordination team when needed. Adherence to guidelines of around 70% was similar in all countries, as well as constant and considerably lower adherence for prophylaxis.

Some criticisms have been published on the implementation of the WHO protocol,^24^ such as, for example, lack of information in medical records, misclassification of patients in relation to wards and type of infections (e.g. definition of HAIs) and a low acceptability of staff to perform the PPS. In our experience, the main challenges faced were related to the categorization of the type of indication (e.g. between *Other* and *Unknown*) and for the accuracy of the diagnosis (e.g. *Undefined* or *Unknown*). In both situations, this difficulty was associated with a lack of information in some cases, but mostly with the somewhat confusing definitions in the original protocol. These issues prompted several reviews both with hospital coordinators and for the final analysis of results. On the other hand, the strong support from hospital authorities to conduct the study, the careful selection of the research team by the hospital coordinators and their intensive training, and the collaboration of the attending physicians of each ward on the day of the survey considerably facilitated the development of the study.

The study has some limitations. Firstly, despite being the largest study performed in Latin America, it is still a sample of hospitals selected by the MoHs and universities, not representing the entire hospital population of every country. Secondly, comparisons with other studies is limited by several factors, such as the type and complexity of hospitals included, the methodology for the hospital selection, the sample size, the possible temporal variations in antibiotic use (e.g. due to changes in resistance patterns) and, probably more importantly, some differences in data collection methods and in the overall process for assessing the guidelines compliance.

Main strengths of this study are the largest (so far) number of countries and hospitals in the region and the data quality control. The latter was achieved through (i) intensive training of the hospital research teams; (ii) continuous communication and technical support; and (iii) permanent review of data inconsistencies during the survey period. Additionally, the adoption of a flexible data collection tool allowed the implementation of the survey even in facilities with human resource and IT constraints.

### Conclusions

This study shows that there are key elements that should be addressed as a priority by MoHs, professional associations and regional organizations promoting ASPs. It is essential to develop and/or strengthen these programmes considering both a diagnostic approach (including a PPS if feasible) and attitudinal aspects related to antimicrobial prescribing. Emphasis should be placed on the implications for AMR of inappropriate prescribing, the need to improve compliance with CPGs, especially for surgical prophylaxis, and on establishing antibiotic selection criteria according to each indication (e.g. avoiding 3GCs as the initial choice for CAIs and surgical prophylaxis, reserving carbapenems to treat selected HAIs). It is also critical to increase microbiological diagnostics (e.g. by improving the access to diagnostic tools, increasing the submission of samples to the laboratory, and using the results to tailor the treatment). To achieve all these goals, it is necessary to ensure continuous and structured education for prescribers. In this regard, in November 2020, PAHO launched an e-learning training course for the ‘Implementation and Strengthening of Antimicrobial Stewardship Programs’, which is currently active at the time of this publication.

After completing the PPS, and under the umbrella of MoHs, first steps to implement an ASP (e.g. a baseline evaluation of human resources involved and previous activities related to antimicrobial stewardship initiatives, conformation of ASP teams, and sharing of PPS results with prescribers) began in many participating hospitals. Shortly afterwards, the COVID-19 pandemic struck, leading to serious difficulties in continuing with the progress of the programmes, due to the scarcity of human and material resources, and the redistribution of the tasks of most of the actors involved. At the time of publication of this study, as assessed in meetings with many of the participating hospitals, many of them have been progressively resuming ASP activities.

## Supplementary Material

dkab459_Supplementary_Data

## Supplementary data

Tables S1 to S4 and Appendix 1 are available as Supplementary data at *JAC* Online.
