# Supplementary material for: Point prevalence survey of antibiotic use in hospitals in Latin American countries
Source: J Antimicrob Chemother. 2021 Dec 27;77(3):807–15. doi: 10.1093/jac/dkab459 (PMC9092443; doi:10.1093/jac/dkab459)
Supplement: dkab459_Supplementary_Data [file dkab459_supplementary_data.docx]

**Supplementary data**

**Table S1. Antibiotics prescribed for community-acquired infections (CAIs) in the Latin America point-prevalence survey, 2018–2019**

|  | **Cuba** | | **Mexico** | | **El Salvador** | | **Peru** | | **Paraguay** | | **Total** | |
| --- | --- | --- | --- | --- | --- | --- | --- | --- | --- | --- | --- | --- |
| **Antibiotic group** | **n** ^a^ | **%** | **n** | **%** | **n** | **%** | **n** | **%** | **n** | **%** | **n** | **%** |
| J01DD Third-generation cephalosporins (ceftriaxone, cefotaxime, ceftazidime) | 143 | 34.1 | 69 | 30.4 | 236 | 31.6 | 157 | 29.7 | 74 | 25.2 | **679** | **30.6** |
| J01MA Fluoroquinolones (ciprofloxacin, levofloxacin) | 31 | 7.4 | 23 | 10.1 | 95 | 12.7 | 35 | 6.6 | 31 | 10.5 | **215** | **9.7** |
| J01DH Carbapenems (meropenem, imipenem, ertapenem) | 9 | 2.1 | 30 | 13.2 | 64 | 8.6 | 77 | 14.6 | 6 | 2.0 | **186** | **8.4** |
| J01XD Imidazole derivatives (metronidazole) | 32 | 7.6 | 26 | 11.5 | 61 | 8.2 | 42 | 7.9 | 20 | 6.8 | **181** | **8.2** |
| J01FF Lincosamides (clindamycin) | 5 | 1.2 | 16 | 7.0 | 54 | 7.2 | 55 | 10.4 | 31 | 10.5 | **161** | **7.3** |
| J01CR Combinations of penicillins, including beta-lactamase inhibitors (amoxicillin-sulbactam, piperacillin-tazobactam, amoxicillin-clavulanic acid, ampicillin-sulbactam) | 19 | 4.5 | 9 | 4.0 | 37 | 4.9 | 13 | 2.5 | 58 | 19.7 | **136** | **6.1** |
| J01GB Other aminoglycosides (gentamicin, amikacin) | 24 | 5.7 | 10 | 4.4 | 32 | 4.3 | 27 | 5.1 | 13 | 4.4 | **106** | **4.8** |
| J01XA Glycopeptide antibacterials (vancomycin) | 9 | 2.1 | 14 | 6.2 | 31 | 4.1 | 32 | 6.0 | 6 | 2.0 | **92** | **4.1** |
| J01CA Penicillins with extended spectrum (ampicillin) | 0 | 0.0 | 13 | 5.7 | 42 | 5.6 | 8 | 1.5 | 27 | 9.2 | **90** | **4.1** |
| J01DC Second-generation cephalosporins (cefuroxime) | 72 | 17.2 | 0 | 0.0 | 0 | 0.0 | 4 | 0.8 | 0 | 0.0 | **76** | **3.4** |
| J01FA Macrolides (azithromycin, claritromycin) | 16 | 3.8 | 2 | 0.9 | 13 | 1.7 | 18 | 3.4 | 10 | 3.4 | **59** | **2.7** |
| J01EE Combinations of sulfonamides and trimethoprim, includingderivatives (trimethoprim-sulfamethoxazole) | 19 | 4.5 | 1 | 0.4 | 9 | 1.2 | 13 | 2.5 | 0 | 0.0 | **42** | **1.9** |
| J01DB First-generation cephalosporins (cefazolin, cephalexin) | 13 | 3.1 | 1 | 0.4 | 4 | 0.5 | 14 | 2.6 | 9 | 3.1 | **41** | **1.8** |
| J01CF Beta-lactamase resistant penicillins (oxacillin, dicloxacillin) | 0 | 0.0 | 3 | 1.3 | 8 | 1.1 | 17 | 3.2 | 2 | 0.7 | **30** | **1.4** |
| J01DE Fourth-generation cephalosporins (cefepime) | 17 | 4.1 | 2 | 0.9 | 5 | 0.7 | 2 | 0.4 | 0 | 0.0 | **26** | **1.2** |
| J01CE Beta-lactamase sensitive penicillins (penamecillin) | 0 | 0.0 | 0 | 0.0 | 24 | 3.2 | 0 | 0.0 | 0 | 0.0 | **24** | **1.1** |
| J01CA Penicillins with extended spectrum (amoxicillin) | 4 | 1.0 | 0 | 0.0 | 8 | 1.1 | 2 | 0.4 | 2 | 0.7 | **16** | **0.7** |
| J01BA Amphenicols (chloramphenicol) | 0 | 0.0 | 0 | 0.0 | 2 | 0.3 | 7 | 1.3 | 0 | 0.0 | **9** | **0.4** |
| J01AA Tetracyclines (doxycycline) | 0 | 0.0 | 2 | 0.9 | 4 | 0.5 | 2 | 0.4 | 1 | 0.3 | **9** | **0.4** |
| J01XE Nitrofuran derivatives (nitrofurantoin) | 1 | 0.2 | 0 | 0.0 | 8 | 1.1 | 0 | 0.0 | 0 | 0.0 | **9** | **0.4** |
| Other antibiotics | 5 | (1.2) | 6 | (2.6) | 11 | (1.5) | 4 | (0.8) | 4 | (1.4) | **30** | **(1.4)** |
| **Total** | **419** |  | **227** |  | **748** |  | **529** |  | **294** |  | **2217** |  |

^a^ Total number of antibiotics usedto treat CAIs; some patients received more than one antibiotic.

**Table S2. Antibiotics prescribed for health care-associated infections (HAIs) in the Latin America point-prevalence survey, 2018–2019**

|  | **Cuba** | | **Mexico** | | **El Salvador** | | **Peru** | | **Paraguay** | | **Total** | |
| --- | --- | --- | --- | --- | --- | --- | --- | --- | --- | --- | --- | --- |
| **Antibiotic group** | **n** ^a^ | **%** | **n** | **%** | **n** | **%** | **n** | **%** | **n** | **%** | **n** | **%** |
| J01DH Carbapenems (meropenem, imipenem, ertapenem) | 11 | 6.9 | 32 | 22.1 | 90 | 21.5 | 77 | 28.9 | 13 | 23.6 | **223** | **21.4** |
| J01XA Glycopeptide antibacterials (vancomycin, teicoplanin) | 16 | 10.0 | 29 | 20.0 | 56 | 13.4 | 51 | 19.2 | 18 | 32.7 | **170** | **16.3** |
| J01DD Third-generation cephalosporins (ceftriaxone, ceftazidime, cefotaxime ) | 44 | 27.5 | 11 | 7.6 | 61 | 14.6 | 43 | 16.2 | 8 | 14.5 | **167** | **16.0** |
| J01GB Other aminoglycosides (amikacin, gentamicin) | 8 | 5.0 | 13 | 9.0 | 41 | 9.8 | 16 | 6.0 | 3 | 5.5 | **81** | **7.8** |
| J01MA Fluoroquinolones (ciprofloxacin, levofloxacin) | 15 | 9.4 | 9 | 6.2 | 36 | 8.6 | 17 | 6.4 | 1 | 1.8 | **78** | **7.5** |
| J01CR Combinations of penicillins, including beta-lactamase inhibitors (piperacillin-tazobactam, ampicillin-sulbactam, amoxicillin-sulbactam) | 5 | 3.1 | 7 | 4.8 | 44 | 10.5 | 8 | 3.0 | 9 | 16.4 | **73** | **7.0** |
| J01XD Imidazole derivatives (metronidazole) | 19 | 11.9 | 4 | 2.8 | 30 | 7.2 | 6 | 2.3 | 1 | 1.8 | **60** | **5.7** |
| J01FF Lincosamides (clindamycin) | 1 | 0.6 | 6 | 4.1 | 19 | 4.5 | 16 | 6.0 | 2 | 3.6 | **44** | **4.2** |
| J01DE Fourth-generation cephalosporins (cefepime) | 14 | 8.8 | 8 | 5.5 | 9 | 2.2 | 1 | 0.4 | 0 | 0.0 | **32** | **3.1** |
| J01CA Penicillins with extended spectrum (ampicillin) | 0 | 0.0 | 8 | 5.5 | 4 | 1.0 | 8 | 3.0 | 0 | 0.0 | **20** | **1.9** |
| J01XB Polymyxins (colistin) | 5 | 3.1 | 0 | 0.0 | 0 | 0.0 | 12 | 4.5 | 0 | 0.0 | **17** | **1.6** |
| J01EE Combinations of sulfonamides and trimethoprim, including derivatives (trimethoprim-sulfamethoxazole) | 6 | 3.8 | 5 | 3.4 | 4 | 1.0 | 2 | 0.8 | 0 | 0.0 | **17** | **1.6** |
| J01XX Other antibacterials (fosfomycin, linezolid) | 3 | 1.9 | 1 | 0.7 | 5 | 1.2 | 3 | 1.1 | 0 | 0.0 | **12** | **1.1** |
| J01DB First-generation cephalosporins (cephalothin) | 0 | 0.0 | 3 | 2.1 | 6 | 1.4 | 0 | 0.0 | 0 | 0.0 | **9** | **0.9** |
| J01AA Tetracyclines (doxycycline) | 0 | 0.0 | 4 | 2.8 | 4 | 1.0 | 1 | 0.4 | 0 | 0.0 | **9** | **0.9** |
| J01DC Second-generation cephalosporins (cefuroxime) | 7 | 4.4 | 0 | 0.0 | 0 | 0.0 | 0 | 0.0 | 0 | 0.0 | **7** | **0.7** |
| J01DF Monobactams (aztreonam) | 3 | 1.9 | 0 | 0.0 | 0 | 0.0 | 0 | 0.0 | 0 | 0.0 | **3** | **0.3** |
| J01XE Nitrofuran derivatives (nitrofurantoin) | 0 | 0.0 | 0 | 0.0 | 3 | 0.7 | 0 | 0.0 | 0 | 0.0 | **3** | **0.3** |
| J01CF Beta-lactamase resistant penicillins (oxacillin) | 0 | 0.0 | 0 | 0.0 | 2 | 0.5 | 1 | 0.4 | 0 | 0.0 | **3** | **0.3** |
| Other antibiotics | 3 | (1.9) | 5 | (3.4) | 4 | (1.0) | 4 | (1.5) | 0 | (0.0) | **16** | **1.5** |
| **Total** | **160** |  | **145** |  | **418** |  | **266** |  | **55** |  | **1044** |  |

^a^ Total number of antibiotics used to treat HAIs; some patients received more than one antibiotic.

**Table S3. Antibiotics prescribed for surgical prophylaxis in the Latin America point-prevalence survey, 2018–2019**

|  | **Cuba** | | **Mexico** | | **El Salvador** | | **Peru** | | **Paraguay** | | **Total** | |
| --- | --- | --- | --- | --- | --- | --- | --- | --- | --- | --- | --- | --- |
| **Antibiotic group** | **n** ^a^ | **%** | **n** | **%** | **n** | **%** | **n** | **%** | **n** | **%** | **n** | **%** |
| J01DB First-generation cephalosporins (cefazolin, cephalexin, cephalothin, cefadroxil) | 33 | 34.0 | 14 | 24.6 | 33 | 27.7 | 25 | 43.9 | 40 | 52.6 | 145 | 35.7 |
| J01DD Third-generation cephalosporins (ceftriaxone, cefotaxime) | 17 | 17.5 | 23 | 40.4 | 63 | 52.9 | 11 | 19.3 | 4 | 5.3 | 118 | 29.1 |
| J01XD Imidazole derivatives (Metronidazole) | 14 | 14.4 | 6 | 10.5 | 5 | 4.2 | 4 | 7.0 | 3 | 3.9 | 32 | 7.9 |
| J01DC Second-generation cephalosporins (cefuroxime) | 23 | 23.7 | 1 | 1.8 | 0 | 0.0 | 0 | 0.0 | 0 | 0.0 | 24 | 5.9 |
| J01GB Other aminoglycosides (gentamicin, amikacin) | 6 | 6.2 | 5 | 8.8 | 1 | 0.8 | 4 | 7.0 | 4 | 5.3 | 20 | 4.9 |
| J01MA Fluoroquinolones (ciprofloxacin) | 2 | 2.1 | 3 | 5.3 | 4 | 3.4 | 2 | 3.5 | 4 | 5.3 | 15 | 3.7 |
| J01CR Combinations of penicillins, including beta-lactamase inhibitors (amoxicillin-sulbactam, amoxicillin-clavulanic acid) | 0 | 0.0 | 0 | 0.0 | 2 | 1.7 | 0 | 0.0 | 11 | 14.5 | 13 | 3.2 |
| J01CA Penicillins with extended spectrum (amoxicillin, ampicillin) | 0 | 0.0 | 1 | 1.8 | 2 | 1.7 | 0 | 0.0 | 10 | 13.2 | 13 | 3.2 |
| J01FF Lincosamides (clindamycin) | 0 | 0.0 | 2 | 3.5 | 4 | 3.4 | 6 | 10.5 | 0 | 0.0 | 12 | 3.0 |
| J01XA Glycopeptide antibacterials (vancomycin) | 1 | 1.0 | 0 | 0.0 | 2 | 1.7 | 4 | 7.0 | 0 | 0.0 | 7 | 1.7 |
| J01FA Macrolides (azithromycin) | 1 | 1.0 | 0 | 0.0 | 0 | 0.0 | 0 | 0.0 | 0 | 0.0 | 1 | 0.2 |
| Other antibiotics | 0 | (0.0) | 2 | (3.5) | 3 | (2.5) | 1 | (1.8) | 0 | (0.0) | 6 | 1.5 |
| **Total** | **97** |  | **57** |  | **119** |  | **57** |  | **76** |  | **406** |  |

^a^ Total number of antibiotics used for surgical prophylaxis; some patients received more than one antibiotic.

**Table S4. Guidance category for prescribing antibiotics for treatment in the Latin America point-prevalence survey, 2018–2019**

|  | **Cuba** | | **Mexico** | | **El Salvador** | | **Peru** | | **Paraguay** | | **Total** | |
| --- | --- | --- | --- | --- | --- | --- | --- | --- | --- | --- | --- | --- |
|  | **n** | **%** | **n** | **%** | **n** | **%** | **n** | **%** | **n** | **%** | **n** | **%** |
| **Guidance category ^a^** | 488 |  | 280 |  | 894 |  | 548 |  | 244 |  | **2,454^b^** |  |
| Empirical treatment | 448 | 91.8 | 200 | 71.4 | 698 | 78.1 | 450 | 82.3 | 221 | 90.6 | **2,017** | **82.2** |
| Targeted treatment | 35 | 7.1 | 76 | 27.1 | 192 | 21.5 | 95 | 17.3 | 23 | 9.4 | **421** | **17.2** |
| Not available | 5 | 0.1 | 4 | 1.4 | 4 | 0. | 3 | 0.4 | 0 | 0 | **16** | **0.7** |
|  | | | | | | | | | | | | |
| **Sample taken for microbiologic diagnostic** | 485 |  | 277 |  | 904 |  | 530 |  | 254 |  | **2,450^2^** |  |
| Yes | 95 | 19.6 | 164 | 59.2 | 517 | 57.2 | 240 | 45.3 | 70 | 27.6 | **1,086** | **44.3** |
| No | 386 | 79.6 | 112 | 40.4 | 384 | 42.5 | 208 | 39.2 | 182 | 71.6 | **1,272** | **51.9** |
| Not available | 4 | 0.8 | 1 | 0.4 | 3 | 0.3 | 82 | 15.5 | 2 | 0.8 | **92** | **3.7** |

^a^ Number of antibiotic treatments prescribed in every category out to the total number of indications for antibiotic treatment,

including those prescribed for unknown reasons. Prophylaxis and other indications are excluded.

^b^ Differences between totals are due to missing values for four patients.

**Appendix 1. PPS data collection form**

This is the PAHO/WHO Point Prevalence survey for the use of antibiotics in hospitals.

**Mandatory variables**



Hospital name



No. of data collector (team)



**Patient data**



Date of survey




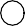
Type of room / unit Pediatric Ward


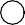
Pediatric surgery Ward


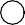
Pediatric High risk Ward


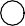
 Pediatric intensive care Ward


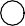
Neonatal intensive care unit


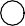
Neonatal Ward


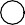
Adult Ward


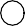
Surgery Ward


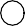
Adults High-risk Ward


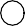
Adult intensive care unit


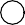
Mixed Ward


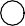
Gynecology and obstetrics

(High-risk units are defined as units or wards that, due to the type of care they provide, have a high consumption of antibiotics. The usual treatment for patients in these high-risk units generally requires antibiotics as part of their care. High-risk units include the following specialties: Hematology, Oncology, Burn Service, Transplantation and Infectious Diseases)



Ward Specialty List of WHO specialties



Name of the Chamber / Unit

National Identification Number (national identity document)




(If the number of the child's record is the same as the mother's, please use the mother's record number and add “IO” at the end. If the national identification number is not available, use the patient's medical record number)



Gender Male Female Transgender Unknown



Date of birth known?
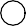
Yes
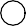
 No



Date of birth (AMD)

Age (years)



___________________________

Age (months) (If less than one year)




Premature birth
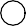
 Yes
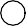
 No
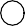
 Unknown



Weight of child (kg)



Date of admission



Was any catheterization performed?
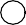
 Yes
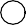
 No
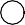
 Unknown



Type of catheterization Urinary
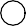
 Peripheral
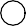
Central
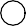
Peritoneal
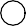
Hemodialysis
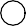
 Other
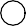
Unknown




Was any intubation performed?
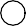
 Yes
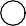
 No
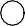
 Unknown



Has the patient undergone any surgical procedure since admission?
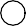
 Yes
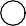
 No
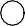
 Unknown

**Optional variables** (The decision to collect the information on the variables included in this section must be made by the national or institutional coordinator or by the main researcher. This decision should not be made individually by the person completing this form)

Does the patient have malaria?
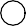
 Yes
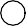
 No
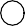
 Unknown



Does the patient have tuberculosis?
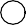
 Yes
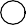
 No
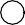
 Unknown



Does the patient have HIV?
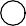
 Yes
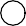
 No
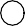
 Unknown



CD4 count in the last 6 months.

(Specify in cells / mm3. If unknown, indicate "Unknown")



Does the patient suffer from malnutrition?
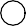
 Yes
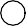
 No
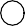
 Unknown



Was the patient transferred from another health center?


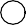
 Yes
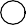
 No
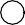
 Unknown



Was the patient transferred from another hospital unit?


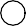
 Yes
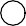
 No
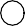
 Unknown



Name of Unit



(Indicate "Unknown" if unknown)



Has the patient been admitted to the hospital where the survey is conducted, within the 90 days prior to current admission?


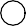
 Yes
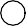
 No
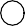
 Unknown



Was the birth the reason for the patient's hospitalization?
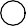
 Yes
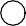
 No
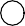
 Unknown

(eg, patients hospitalized for being born in this hospital)



Is the patient currently being given a course of antibiotics other than a TB drug?


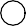
 Yes
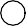
 No

**Indication #1**




**Mandatory Variables**

The patient acquired the infection in ...


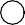
 Health care associated infection (IACS)

Community acquired infection (IAC)

Unknown

Other

Diagnosis of indication No.1 CNS (Central nervous system infections)

EYE ( Endophthalmitis)

NGO (Nose, throat, ear, larynx and mouth infections)

BRON (Acute bronchitis or exacerbation of chronic bronchitis)

NEU (Pneumonia)

CF (Cystic Fibrosis)

CV (Cardiovascular infections: endocarditis, vascular graft)

GI (Gastrointestinal infections ( eg, salmonellosis, antibiotic-associated diarrhea)

IA (intra-abdominal sepsis, including hepatobiliary)

SSI-PTB (Surgical site infection involving skin or soft tissue, but without bone involvement)

O-PTB (Cellulitis, wound, soft tissue deep without bone involvement, not related to surgery)

SSI-A (septic arthritis, osteomyelitis of the surgical site)

OA (septic arthritis, osteomyelitis, not related to surgery)

CIS (Symptomatic infection Lower urinary tract infection (eg, cystitis)

PIE (Symptomatic upper urinary tract infection (eg, pyelonephritis))

BA (Asymptomatic bacteriuria)

OG (Obstetric or gynecological infections, STD in women)

GU (Prostatitis, epididymis-orchitis, STD in men)

BAC (Laboratory confirmed bacteremia)

SEPC (Clinical sepsis: suspected bloodstream infection without laboratory confirmation / no results available, no blood cultures or negative blood culture, excluding febrile neutropenia)

NF (Febrile neutropenia or another form of manifestation of infection in an immunocompromised host (eg. HIV, chemotherapy, etc.) without clear anatomical site)

SIRS (Systemic inflammatory response without clear anatomic site)

IND (Completely undefined; site without systemic inflammation)

NA (Not applicable; for use of antibiotics other than treatment)

Unknown Other

Yes diagnosis appears in the list above, please specify here.

Is it prophylaxis or treatment? Prophylaxis Treatment Unknown

Is it medical or surgical prophylaxis? Medical prophylaxis Surgical prophylaxis Unknown

Duration of surgical prophylaxis One dose

Multiple doses in one day

Multiple doses more than one day Unknown

Is it empirical or targeted treatment? Empirical treatment Targeted treatment Unknown

(empirical means blind therapy; targeted treatment means the antibiotic was chosen or consolidated after culture and sensitivity results)

Start date of the first antibiotic administered for this indication (A MD)

(Day on which administered the first dose of the first antibiotic for this indication)

Was a sample taken for microbiological diagnosis? Yes No Unknown

Type of sample collected Blood

- Urine
- Sputum
- Wound sample
- Other sterile fluids (CSF, Synovial, Peritoneal etc.)
- Other

**Optional variables**

Were the culture results available in the documentation?

Yes No Unknown

Were the results of the bacterial susceptibility tests Yes No Unknown

available in the documentation?

Were the results reported to the treating physician?(This includes Yes No Unknown

oral communication from the laboratory, electronically, by telephone

or printed result, but in any case recorded in

the medical record)

Name of the bacteria isolated in the laboratory

WHO list

Type of antimicrobial

resistance Carbapenem

- resistance Extended spectrum betalactamases ( ESBL)
- Resistance to methicillin
- Resistance to colistin Resistance to vancomycin
- Intermediate resistance to vancomycin
- Resistance to quinolones
- Unknown
- Other resistance No resistance

**Antibiotic #1**

**Mandatory Variables**

Number of the related indication

(Please enter the numerical value of the related indication entered in the previous section. antibiotic cannot be linked to an indication, please enter "No." If the antibiotic is linked to more than one indication, specify the values ​​of the related indications separately (eg 1, 2))

Antibiotic start date (AMD )

Is it a combined product? Yes No

(Combination products are products that contain two antibiotic substances or an antibiotic substance and an enzyme inhibitor)

Name of each antibiotic substance administered

Antibiotic (WHO List)

Antibiotic No2 if it was a combination product (WHO List)

Dose of each active substance administered (only for combined antibiotics)

(The dose of each substance including enzyme inhibitors must be entered in the same order as in the name of the antibiotic in the last question (eg 160mg, 800mg). Enter “No” if no information can be obtained.)

Name of antibiotic in patient record

Unit dose administered to patient

Unit of measurement of unit dose g (grams)

mg (milligrams)

IU (international units)

MU (million international units)

Daily frequency of administration of the unit dose

(eg amoxicillin treatment given every 8 hours. Daily frequency = 24 hours / 8 hours = 3)

Please write your comment here if you are unsure about the calculation.

Route of administration of the antibiotic Oral

Parenteral

Does the prescription of this antibiotic follow any guide or guideline for the indication / diagnosis? (Evaluate whether the choice of antibiotic was in accordance with local guidelines or consult with an infectious disease specialist. If there are no local guidelines, the follow-up of guidelines can be established in comparison with national, regional or international guidelines that will be specified in a timely manner by each hospital. If no guidelines were used or the antibiotic is used for more than one indication, mark as Not evaluable.)

Yes

No

Not evaluable

Insufficient information

**Optional variables**

Is the oral antibiotic the result of a change from the IV route to the oral route?

Yes No Unknown

Type of prescriber Specialist

Responsible physician

Nursing staff

Resident

Reason for the missed dose (if applicable) S: All doses missed due to stock-out

O: No dose due to stock-out

P: Partially, some missed doses due to stock-out,

others for various reasons

U: Unknown

Is there a second antibiotic? Yes No

**Antibiotic #2**

**Antibiotic #3**

**Notes________________________________________________________________________________**

Other comments
